# Supplementary material for: The Health Status of the US Veterans: A Longitudinal Analysis of Surveillance Data Prior to and during the COVID-19 Pandemic
Source: Healthcare (Basel). 2023 Jul 17;11(14):2049. doi: 10.3390/healthcare11142049 (PMC10378995; doi:10.3390/healthcare11142049)
Supplement: Supplementary file 1 [file healthcare-11-02049-s001.zip › healthcare-2464693-supplementary.pdf]

The Health Status of the US Veterans: A Longitudinal Analysis of Surveillance Data Prior to and during the COVID-19 Pandemic

Supplementary Material

Supplemenatary Figure S1. Odds Ratios by Disease by Age Group (excludes Mental Health).

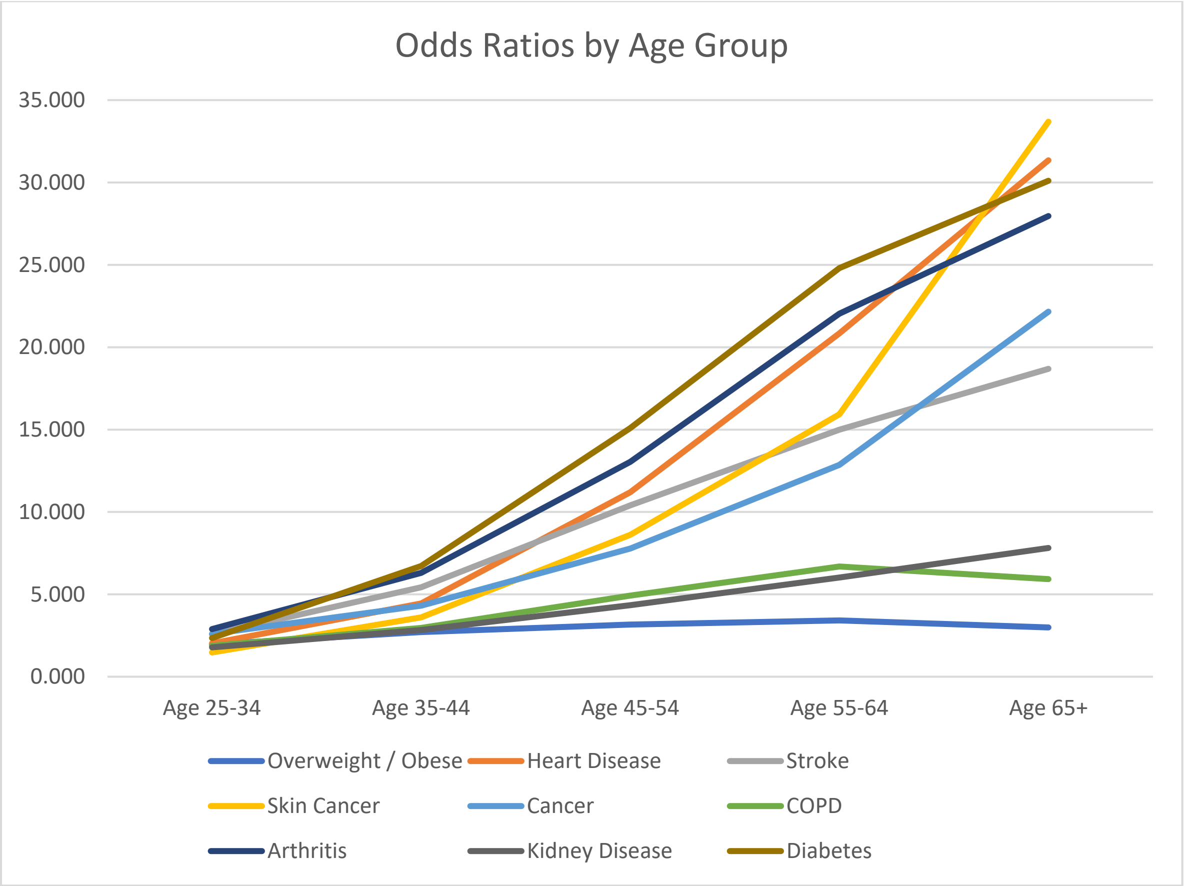

Supplementary Figure S1. Odds Ratios by Disease by Age Group (excludes Mental Health).
